# Long-Time Behavior of Surface Properties of Microstructures Fabricated by Multiphoton Lithography

**DOI:** 10.3390/nano11123285

**Published:** 2021-12-03

**Authors:** Mateusz Dudziak, Ievgeniia Topolniak, Dorothee Silbernagl, Korinna Altmann, Heinz Sturm

**Affiliations:** 1 BAM Bundesanstalt für Materialforschung und -Prüfung, Unter den Eichen 87, 12205 Berlin, Germany; dorothee.silbernagl@bam.de (D.S.); korinna.altmann@bam.de (K.A.); Heinz.Sturm@bam.de (H.S.); 2 Institute for Machine Tools and Factory Operations (IWF), TU Berlin, Pascalstr. 8-9, 10587 Berlin, Germany

**Keywords:** multiphoton lithography, microfabrication, SZ2080, Young’s Modulus, aging, surface properties, X-ray photoelectron spectroscopy, atomic force microscopy, force distance curves

## Abstract

The multiphoton lithography (MPL) technique represents the future of 3D microprinting, enabling the production of complex microscale objects with high precision. Although the MPL fabrication parameters are widely evaluated and discussed, not much attention has been given to the microscopic properties of 3D objects with respect to their surface properties and time-dependent stability. These properties are of crucial importance when it comes to the safe and durable use of these structures in biomedical applications. In this work, we investigate the surface properties of the MPL-produced SZ2080 polymeric microstructures with regard to the physical aging processes during the post-production stage. The influence of aging on the polymeric microstructures was investigated by means of Atomic Force Microscopy (AFM) and X-ray Photoelectron Spectroscopy (XPS). As a result, a time-dependent change in Young’s Modulus, plastic deformation, and adhesion and their correlation to the development in chemical composition of the surface of MPL-microstructures are evaluated. The results presented here are valuable for the application of MPL-fabricated 3D objects in general, but especially in medical technology as they give detailed information of the physical and chemical time-dependent dynamic behavior of MPL-printed surfaces and thus their suitability and performance in biological systems.

## 1. Introduction

Three-dimensional printing technologies have been gaining popularity for several decades due to their versatility, ease of use, and affordable prices. Nowadays, the most commonly used photoinduced 3D printing methods are Inkjet printing, Stereolithography (SLA), and Digital Light Processing (DLP) [1,2,3]. Despite the wide range of applications and inherent advantages of each technique, they still struggle with limited manufacturing control and insufficient resolution [4,5,6], often leading to objects with significant fragility [7,8]. The Multiphoton Lithography (MPL) technique has been shown to overcome most of these drawbacks. This state-of-the-art technique is based on the non-linear absorption of two or more photons, which enables polymerization in a small volume (voxel) of the photosensitive matter [9]. MPL is accurate and detailed due to its fine control of the fabrication process and non-linear nature, resulting in spatial resolution in the order of 0.1–1 μm [10]. This permits MPL to produce structures ranging in size from submicron [11] up to hundreds of micrometers [12,13]. Even higher pattern resolution can be achieved by applying the Stimulated Emission Depletion (STED) technique [14,15]. Currently, MPL is widely used in applications such as biotechnology [16], microelectronics [17], the semiconductors industry [18], and micro-optics [19,20]. Many organic, as well as inorganic–organic, hybrid materials are used as monomers [3]. The systems based on radical polymerization are (meth)acrylate-based [21], and thiol–ene and thiol–yne systems [22]. The addition of β-allyl sulfones [23,24], as well as cationic systems, including azahelicenes, SU-8 or SCR-701, is also employed in MPL [25,26,27].

Applying NIR femtosecond laser in MPL makes it compatible with biological samples as this treatment is harmless to living research objects, including cells [28]. An example of using MPL technologies in bioengineering is presented by Koroleva et al. Scaffolds made of four-armed polylactic acid showed biocompatibility with human neural tissues. Moreover, it was evidenced that this photocurable material not only is non-toxic to neuronal cells, but also does not interfere with their proliferation and growth [29]. Therefore, this technique finds broad usage in the field of biophysics and biosystems. For instance, Psycharakis et al. used hybrid materials based on titanium and zirconium, and methacrylic acid as crosslinkable ligands to produce cellular scaffolds employing MPL. They showed that it is not only the chemistry of material but also the shape and layout of the structure that are crucial. The porosity of the printed material is extremely important in biological research as it allows for convenient cell proliferation, as well as their free expansion over the entire surface of the material [30]. Nevertheless, despite extensive investigations of 3D materials for biomedical application, time-dependent surface properties of MPL-fabricated microstructures have received very little attention. Commonly assumed to be constant with time, this important parameter is critical when it comes to the material application lifespan and dynamics of the polymer interactions with the applied biological environment.

Since surface properties, both physical and chemical, are crucial in the fields of bioengineering, the interaction of material and living matter has a significant impact on their applicability and suitability in bio-med systems. One of the strategies, as mentioned above, is chemical functionalization of the surface of MPL microstructures in order to achieve the desired performance. In this work, we report the details on surface aging, which should be taken into consideration for future approaches to alter microstructure properties and thus biocompatibility.

SZ2080 organic–inorganic hybrid photoresist is widely used in MPL due to its unique crosslinking properties [31]. It exhibits extra-low shrinkage during polymerization, resulting in negligible distortion of the printed structure [32]. The pre-clinical studies on MPL-fabricated SZ2080 microstructures demonstrated great biocompatibility of the scaffolds comparable to commercially available collagen membranes [33]. Being promising in the development of high-precision biocompatible structures, SZ2080 was selected for further investigation in the scope of this work.

Space-resolved characterization of the MPL microstructures is challenging and requires high-resolution methods with low material consumption. Among those are Atomic Force Microscopy (AFM) [34], X-ray Photoelectron Spectroscopy (XPS) [35], Transmission Electron Microscopy [36], Scanning Electron Microscopy (SEM), Small- and Wide-angle X-ray Scattering [37], and Flash Scanning Calorimetry [38]. To measure local mechanical properties of microstructures, AFM was employed to investigate polymers and nanocomposite surfaces [39], including MPL-structured material [40,41,42]. The measured volumes of mechanical measurements by means of AFM are typically in the range of 1 µm^3^ or smaller, due to the small contact area between the AFM tip and applied forces that are in the range of µN during contact [43,44] and can be used to determine micromechanical properties of materials such as their Young’s Modulus, plastic deformation, and adhesion [45].

Herein, we focus on the characterization of surface properties of the SZ2080 polymer microstructures fabricated with the MPL technique. XPS and AFM force–distance curves (FDC) were used to monitor chemical and mechanical properties, respectively. The samples were examined during the time span of 25 days to determine physical and chemical changes at the surface. The obtained results on the time-dependent surface behavior of polymeric microstructures unraveled the guidelines for the reproducible and well-defined utilization of polymeric structures fabricated with MPL technique.

## 2. Materials and Methods

### 2.1. Materials and Sample Preparation

SZ2080 negative photoresist (IESL-FORTH, Heraklion, Greece) containing 1 wt% of Michler’s ketone photoinitiator was used in this work for MPL fabrication. A drop of the above-mentioned material was applied on the glass slip and heated, first at 60 °C for one hour, and afterwards at 100 °C for an additional hour. Next, a 3D microstructure was fabricated within the photoresist drop by applying MPL. After the fabrication process, the sample was immersed in the methyl isobutyl ketone (MIK, Sigma Aldrich, Taufkirchen, Germany) for one hour to remove the residue of unpolymerized material and then rinsed with the fresh portion of solvent a couple of times. The MIK solvent was used without prior purification. Finally, the obtained microstructure was dried in air and kept in the dark prior to further analysis at constant temperature and a relative humidity of 22 °C and 31%, respectively.

### 2.2. Methods

#### 2.2.1. Multiphoton Lithography

The 3D microstructures were produced with Laser Nano Factory (Femtika Ltd., Vilnius, Lithuania) and equipped with an Erbium-doped fiber laser (Menlo, Martinsried, Germany) emitting at 780 nm with a repetition rate of 100 MHz for the used 100 fs pulses. Beam focusing was performed by applying an oil-immersion objective with 1.4 numerical aperture (Plan Apochromat 63×, ZEISS, Jena, Germany). An array of repetitive and identical 30 × 30 × 30 µm³ cubic structures was produced for AFM analysis. Structures of 2000 × 1000 × 5 µm³ were fabricated for further characterization with XPS. All structures were obtained by applying the laser power and scanning velocity of 12 mW and 7000 μm/s, respectively. The hatching and slicing distances were set at 0.2 µm and 0.5 μm, respectively. Hatching direction alternated in x-y directions with each layer. with each layer.

#### 2.2.2. Force Distance Curves Atomic Force Microscopy

Atomic force microscopy (AFM) measurements were performed with an MFP-3D microscope (Oxford Instruments Asylum Research, Santa Barbara, CA, USA). The AFM was equipped with a Pointprobe NCL cantilever (Nanosensor, Wetzlar-Blankenfeld, Germany) with an elastic constant of k_c_ = 51.8 N/m and a Si tip with a radius of approximately R = 20 nm, which was found by reference measurements on glass, which was assumed to have a Young’s Modulus of E_glass_ = 72 GPa, a Poisson’s ratio of ν = 0.3, and a resonant frequency f_res_ = 340.8 kHz.

Force volume mode was used with a cantilever deflection trigger of 100 nm [46]. In addition, 10 × 10 FDC indentations were recorded on 10 μm^2^ in the centric area of the microstructure’s top surface, to be averaged for a better signal-to-noise ratio and to compensate for inhomogeneities of the surface [45]. The indentation frequency used is 1 Hz.

AFM measurements were performed after one day post-fabrication and then carried out repeatedly until day 25. For each measurement, an undeformed cube of the same batch was used. For reference purposes, glass was tested after each measurement of the sample in order to control both the geometry and the condition of the tip. Measurements on glass were found to be highly reproducible for the duration of the experiment, and hence the tip remained uncontaminated and sharp, as shown in Figures S1 and S2. All measurements were performed at room temperature.

**Figure 1:**
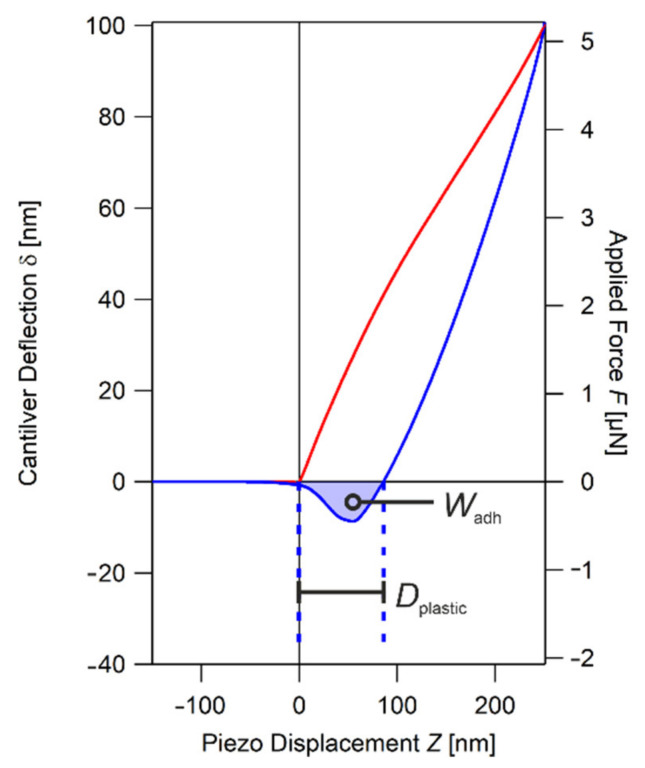
Averaged Force Distance Curve from MPL-fabricated SZ2080 microstructure at 25th day of post-fabrication. The red line corresponds to the approach part of the curve and the blue line to the retract part. The plastic properties are quantified by the plastic deformation D_plastic_ and work of adhesion W_adh_.

From the averaged FDC, the overall mechanical behavior can be investigated, since both elastic and plastic deformations are recorded. In order to quantify plastic properties, typical features of the FDC are examined. In plotting the approach (red) and retract (blue) parts of the FDC in Figure 1, the large hysteresis is clearly visible. The reason for the hysteresis is that the sample stays deformed even after the tip and sample are detached, since it has been plastically deformed. As a measure for this property, we take the indent’s depth D_plastic_, which is the difference between the height of the surface at approach (Z(δ_approach_ = 0) ≡ 0), and the height of the surface at retract (Z(δ_retract_ = 0)). The plastic deformation of the sample reflects its softness, and hence the stability of the printed structure under loads. With softness in most polymeric materials also comes the ability to adhere to other surfaces. It can be quantified in the work of adhesion W_adh_, which is the work needed to detach the AFM probe from the sample surface. For that, the applied force F is calculated from Hooke’s law:

*F* = *k_c_δ* (1)

with *k_c_* being the spring constant of the cantilever and *δ* the deflection of the cantilever. In the plot of applied force *F* versus piezo displacement Z, the work of adhesion W_adh_ corresponds to the area of the jump off contact (JOC) of the retraction (blue curve), as shown in Figure 1.

In order to determine the elastic properties of the printed structures, the deformation has to be calculated as follows:

*D* = *Z* − *δ* (2)

The reduced Young’s Modulus E* can be now assessed by using the Hertz model:

(3) $$D={\left(\frac{F}{\sqrt{R}E^{*}}\right)}^{\frac{2}{3}}$$

where *D* is the deformation, *F* is the applied force, and *R* is the tip radius.

Further, knowing the properties of the AFM tip and the reduced Modulus, the Young’s Modulus can be determined by the following equation:

(4) $$\frac{1}{E^{*}}=\frac{3}{4}\left(\frac{1-\nu_{tip}^{2}}{E_{tip}}+\frac{1-\nu^{2}}{E}\right)$$

where *ν_tip_* is the Poisson’s ratio of the tip (0.33), *ν* is the Poisson’s ratio of the sample, *E_tip_* is the Young’s Modulus of the tip (225 GPa), and *E* is the Young’s Modulus of the sample. This model is only valid for elastic deformations [45,47].

As mentioned above, both elastic and plastic responses of the material are recorded by FDC. The elastic deformations are present at small applied forces; therefore, the Hertzian fit (Equation (3)) can be applied to the approach part of the FDC in order to calculate the Young’s Modulus. At a certain mechanical load, F_yield_, the material starts to yield and deforms irreversibly. The yield force F_yield_ is an important indicator for the mechanical stability of the material and thus is additionally assessed for each measurement, as shown in the Supplementary Materials (Figure S3).

#### 2.2.3. X-ray Photoelectron Spectroscopy

X-ray Photoelectron Spectroscopy (XPS) analysis was performed with a spectrometer SAGE 150 (Specs, Berlin, Germany) equipped with a hemispherical analyzer Phoibos 100 MCD-5. The pressure in the analysis chamber was about 3 × 10^−7^ Pa, and non-monochromatic AlKα radiation was used. The X-ray source is located at a 54.9° angle to the lens system of the analyzer with the analyzer at 18° to the surface at a vertical 90°. Identically prepared, freshly fabricated, and 25-day-aged samples were measured horizontally at 0° in order to detect chemical changes that appeared during aging. In addition, the freshly printed sample was analyzed at three different flatter entry angles of the X-rays (43.7°, 53.7°, and 58.4°), thus giving the information at shallower depths from the polymer surface. XPS spectra were collected in constant analyzer mode. The size of the measurement spot was about 1 × 3 mm², which is slightly larger than the pure sample area of 2000 × 2000 × 5 µm³ but not problematic due to the titanium sample holder. Evaluation of the measured results occurred using CasaXPS. The C1s (as shown in Supplementary Figure S4) peak was standardized to the C-C peak at 285 eV and deconvoluted. All C1s spectra were fitted according to the characteristic binding energies of C-C (285 eV), C-O (286.5 eV), C=O (288 eV), and O-C=O (289 eV).

#### 2.2.4. Scanning Electron Microscopy

Material surface was analyzed with a Zeiss EVO MA 10 (Carl Zeiss Microscopy GmbH, Jena, Germany) scanning electron microscope. The secondary electron mode with an acceleration voltage of 10 kV was used to obtain the images.

## 3. Results and Discussion

### 3.1. Topography of MPL Structures

**Figure 2:**
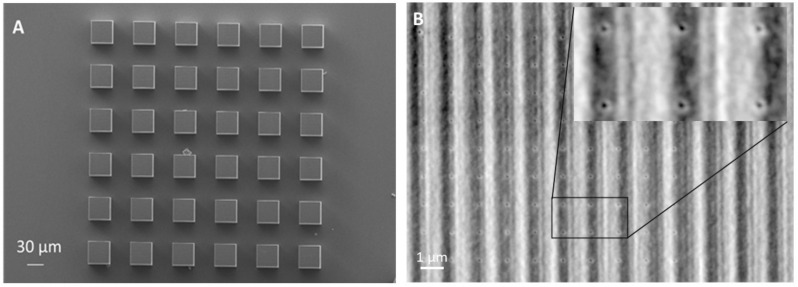
SEM micrographs of SZ2080 MPL-printed cubic structures used for time-dependent AFM investigation: (**A**) Array of identical cubes fabricated at the same MLP parameters. Each cube was used for only one AFM FDC measurement on a given day to avoid effect of the resulted structure artifacts on the further measurements. (**B**) Magnified surface of one of the cubes after AFM FDC measurement. Observed vertical line-like patterns are the result of printing procedure and correspond to laser scanning direction. Pointwise plastic deformations observed in a repetitive manner are caused by AFM FDC measurements.

The accuracy of the fabrication can be observed in the SEM micrographs of SZ2080 cubic microstructures after AFM FDC analysis (Figure 2). Vertical and horizontal linear features that were produced by laser beam movement path can be detected. The print has been made in a repeatable manner.

As can be seen in the SEM micrograph (Figure 2B), where the AFM indents are visible, FDC curves were taken on the sample surface with the tested area of 10 × 10 μm. By averaging approximately 100 curves per measurement, it was assured that the topography artifacts and possible inhomogeneities, when present, do not contribute to the outcome of the data analysis.

### 3.2. Mechanical Properties of the Surface

**Figure 3:**
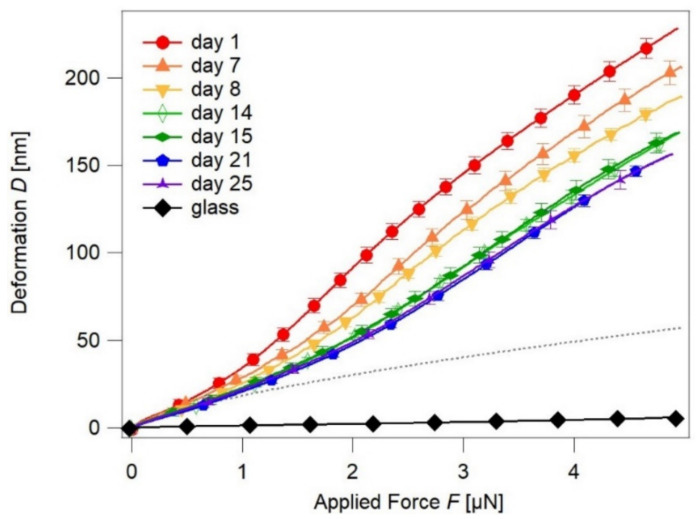
Mechanical properties of SZ2080 microstructures: material deformation depending on the applied force. Aging in the dark at the ambient temperature. The grey dotted line is an exemplary Hertz fit (Equation (3)) with E = 1.3 GPa and R = 21 nm, which only describes the elastic deformation. The experimental data show plastic deformation for forces F > F_yield_, where the Hertz fit cannot describe the deformation.

The first FDC map was recorded 24 h after the SZ2080 structure was produced. Further, the series of FDC curves was recorded over the following 25 days and the corresponding D_plastic_, W_adh_, F_yield_, and Young’s Moduli E of the surface were calculated. Figure 3 represents the detected changes in the sample deformation with respect to the applied force.

One can observe that the maximum deformation D_max_ of the SZ2080 gradually decreases with the aging time and then stabilizes from the 14th day on. Measurements done at days 14 and 15 show the same results in the margin of error. One can then assume that changes of mechanical properties slow down with time. Therefore, the intervals between measurements were increased until properties stabilized, as can be seen for the results from days 21 and 25. The decrease in deformation rate over time is supported by an increase in the Young’s Modulus. The Young’s Modulus is assessed by Equation (3) for D(F = 0) < D < D(F_yield_), with values rising by two times from 0.775 up to 1.3 GPa (Table 1). These values are in very good agreement with the previously published mechanical properties of SZ2080 (0.6–1.1 GPa) [48]. However, in the mentioned work, the effect of time on the mechanical properties was not pursued. The results in this study indicate a drastic change of the elastic properties of the SZ2080 microstructure surface during the first three weeks within the post-fabrication time.

**Figure 4:**
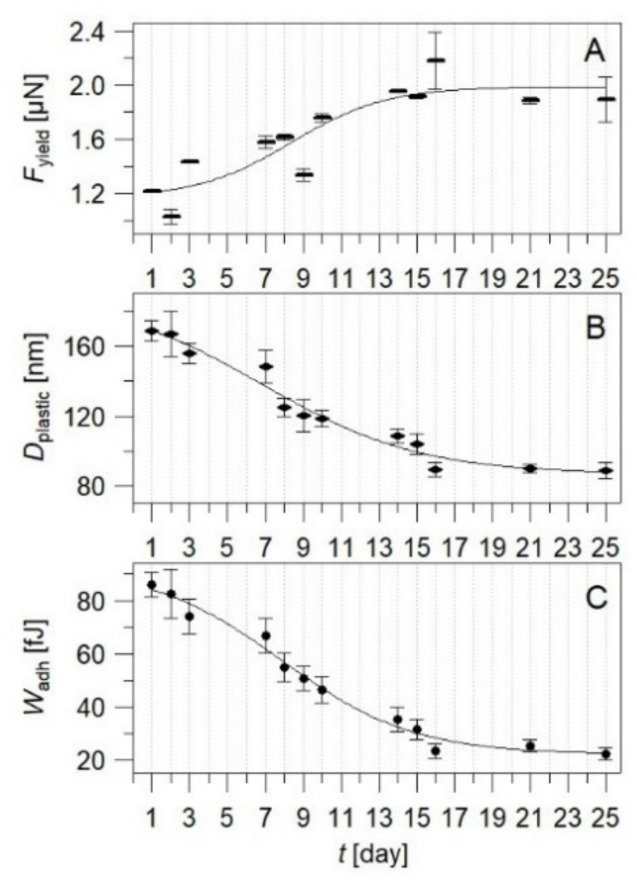
Mechanical properties of SZ2080 microstructures as a function of post-fabrication time: (**A**) mechanical load (*F*_yield_); (**B**) plastic deformations (*D*_plastic_) resulting from maximum applied force of F_max_ = 52 µN; (**C**) tip adhesion (*W*_adh_) to the surface of measured structure. The black solid line is a sigmoidal fit for each data set.

Further FDC analysis shown in Figure 4 identified mechanical load for plastic deformations F_yield_, plastic deformation D_plastic_, and tip adhesion W_adh_ in dependence of the post-fabrication time. An increase in F_yield_ of the polymer on consecutive days is observed in Figure 4A. As represented, from day one until day 14, the amount of the force increases and, afterwards, does not significantly change, indicating a plateau region. Meanwhile, D_plastic_ of SZ2080 exhibits the reversed trend over the time (Figure 4B). As a result, one can observe a noticeable decrease in the plastic strain from 169 nm down to 88 nm for the first hours after printing and to the final days of the measurement, respectively. This implies that, during the first 14 days of experiment, the surface of the sample is more susceptible to plastic deformation that may indicate the softness and lower crosslinking degree of methacrylate groups.

We also observe that the AFM tip adhesion (Figure 4C) shows a similar tendency with the post-fabrication time as D_plastic_. The W_adh_ decreases during the first 16 days from 86 to 27 fJ and, afterwards, remains in the range of 22–27 fJ. This indicates that the specimen undergoes significant changes of the surface, leading to a more constant nature of the surface properties and inertness towards the environment that is indicative of a loss of plasticity in favor of elasticity.

In order to quantify the post-fabrication time dependence of the plastic properties of SZ2080, a sigmoidal fit was applied to the data sets shown in Figure 4 (F_yield_, D_plastic_, and W_adh_). From the sigmoidal fit, a conversion degree was calculated following the probability distribution of a bell curve, which is the derivative of a sigmoid (shown in Figure S5). The resulting values are gathered in Table 2. As one can see, after 14 days F_yield_ has stabilized with 97.7% of the plateau value. However, plastic deformation and adhesion stabilize a little bit slower, reaching 97.7% of their final value after 19 and 18 days, respectively.

Concluding from AFM FDC measurements, the surface of SZ2080 has shown dynamic behavior over time. The first 14–16 days after printing can be characterized by drastic changes in F_yield_, D_plastic_, W_adh_, and Young’s Modulus E. On the other hand, surface stabilization with a furthered aging period is observed and indicates a more inert stage of microstructure surface.

### 3.3. Chemical Composition of SZ2080 Surface

**Figure 5:**
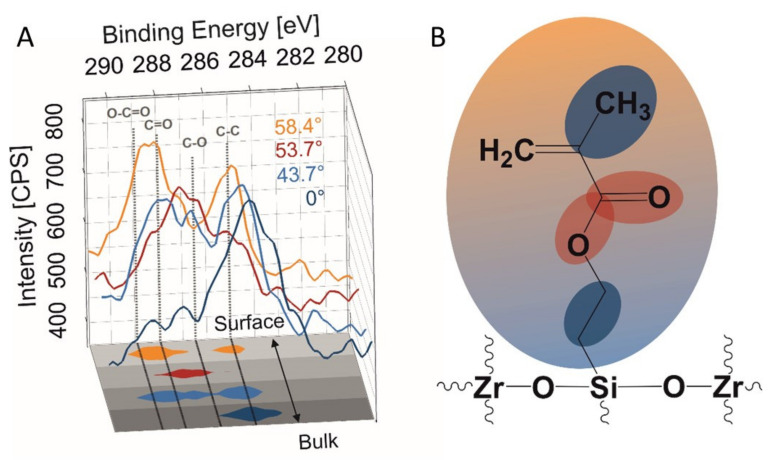
(**A**) Spatial representation of the C1s peak XPS spectra of different measurement angles (yellow 58.4°, red 53.7°, light blue 43.7°, and blue 0°). Peak areas (detected at Intensities > 635 CPS) are projected on a scheme of sample depth (bottom of 3D plot). (**B**) Abstracted scheme of the surface composition as a function of the information depth of the fresh sample SZ2080. The selected colored ellipses represent the chemical groups detected by XPS.

To better understand chemical changes underlying the observed stabilization of SZ2080 surface, MPL-fabricated samples were analyzed by means of XPS within 24 h after fabrication (initial state) and again once they reached 25 days (stabilized state) of post-fabrication time. One should keep in mind that XPS information typically corresponds to the first 5–7 nm of structure surface [49]. To access information at different penetration depths within the top 7 nm of the surface, additional angle-dependent measurements of the freshly prepared sample were performed, whereby the sample was placed at 43.7°, 53.7°, and 58.4° from the X-ray source and compared to 0° (horizontally flat) standard measurement. The obtained results are presented in Figure 5A and Supplementary Materials (Figure S5).

Deconvolution of the spectra of the fresh sample (0° measurement, Figure S4) resulted in 85.2% for C-C and C-O groups of all the C1s. Meanwhile, the carbonyl and carboxyl peaks were measured to be 6.7% and 8.1%, respectively. This indicates oxidized hydrocarbons on the surface that probably come from aging processes in the contact of the surface with air. The chemical composition and molecular configuration of the SZ2080 structure is very complex; however, the simplified structure is suggested for ease of XPS data interpretation (Figure 5B). With an increase in the measurement angle (reduced information depth), the carbonyl peak increases from 20.3% (43.7°) to 54.2% (58.4°). At the same time, the carboxyl peak rises from 20.3% (43.7°) to 23.9% (53.7°) and decreases to 5.4% at a 58.4° measurement angle. That suggests a layer-like distribution of functional groups with the distance to the surface.

Most chemical changes due to aging are to be expected on the surface in the area of the SZ2080 organic functional groups. To investigate this effect, the C1s peaks of the freshly printed microstructure and the one after 25 days of post-fabrication time were compared by performing horizontal (0°) measurement. The obtained results are summarized in Table 3. Comparison of the peaks corresponding to the binding energies of C-C, C-O, C=O, and O-C=O indicates the chemical modifications at the polymer structure with the increase in post-fabrication time. A significant decrease in the C=O peak intensity with time signifies the post-curing process, and hence explains a decline in the surface adhesion observed with the AFM technique. In addition, an increase in C-O peak intensity from 1.6 to 12.9% is observed, whereas the peak corresponded to the C-C bond decreases in intensity by 6.2%. Overall, the obtained results refer to two processes: on the one hand, further crosslinking of the molecules takes place through post-curing, and on the other hand, oxidation of the surface occurs. An increase in C-O amount in the SZ2080 surface structure can explain a decline in its adhesive properties during interactions with the AFM tip.

## 4. Conclusions

In summary, the time-dependent surface behavior of MPL-fabricated SZ2080 microstructures was investigated by means of AFM, FDC, and XPS. Consequently, the information on correlating Modulus, surface yield, adhesion, and chemical surface composition has been presented in this work. The observed process of surface aging during the post-fabrication stage is defined by both mechanical and chemical changes. It was observed that the tip adhesion and plastic deformation decreased, while mechanical load and Young’s Modulus increased during the first 14–16 days. In the period after 16 days, the physical properties of the surface stabilized, indicating final enhancement of the SZ2080 surface. The observed phenomena were underlined by chemical changes at the surface due to oxidation processes and crosslinking. To successfully apply SZ2080 microstructures, it is beneficial to employ printed structures at least 14 days after their fabrication to avoid disadvantageous interactions of the active groups of polymer surface with biological systems. Therefore, we suggest that structures with a post-fabrication time over 16 days would exhibit better properties in terms of biocompatibility. The approach proposed in this work might be another solution to expand the applicability of SZ2080.

## Figures and Tables

**Table 1:** Young’s Moduli of the SZ2080 microstructures with respect to the post-fabrication time.

Post-Fabrication Period [Days]	Young’s Modulus *E* [GPa]
1	0.775 ± 0.002
7	0.94 ± 0.04
8	1.05 ± 0.02
14	1.21 ± 0.006
15	1.24 ± 0.01
25	1.3 ± 0.1

**Table 2:** Conversion degree of plastic mechanical properties of the SZ2080 microstructures with respect to the post-fabrication time in days. Following the integrated form of the empirical rule, the mean value µ equals 50% conversion degree and adding multiples of the standard deviation σ equals 84.1, 97.7, and 99.8% conversion degree.

	µ 50%	µ + σ 84.1%	µ + 2σ 97.7%	µ + 3σ 99.8%
*F*_yield_	8.4	12.2	14	19.8
*D*_plastic_	6.9	12.9	18.9	24.9
*W*_adh_	7.9	13	18.1	23.2

**Table 3:** Percentage content of C1s-related chemical groups obtained from XPS spectra for SZ2080 microstructure at different post-fabrication times.

Functionality	C-C	C-O	C=O	O-C=O
Binding energy [eV]	285.0	286.5	288.0	289.0
Day 1 [%]	83.6	1.6	6.7	8.1
Day 25 [%]	77.4	12.9	1.8	7.9

## Supplementary Materials

The following are available online at https://www.mdpi.com/article/10.3390/nano11123285/s1, Figure S1. Averaged FDC from reference measurements on glass: curves are highly reproducible throughout the whole experiment and in very good agreement with the Hertzian fit. Figure S2. Comparison of work of attractive forces during approach Wattr of glass and SZ2080 over time. As attractive forces of glass are fairly stable, indicating a clean tip with unchanged geometry, attractive forces of SZ2080 increase, an indication of increased density of the material at the surface of MLP-fabricated structures. Figure S3. Fyield is determined by the point of intersection of linear fits of the deformation D3/2 in the regime of elastic and plastic deformations. Figure S4. Deconvoluted representation of peaks of C1s for freshly printed sample at day 1 (A) and aged sample day 25 (B). Figure S5. Statistical analysis, including the bell curve with its characteristic areas µ and µ + (n*σ).
