# Supplementary material for: Long-Time Behavior of Surface Properties of Microstructures Fabricated by Multiphoton Lithography
Source: Nanomaterials (Basel). 2021 Dec 3;11(12):3285. doi: 10.3390/nano11123285 (PMC8708078; doi:10.3390/nano11123285)
Supplement: Supplementary file 1 [file nanomaterials-11-03285-s001.zip › nanomaterials-1467991-supplementary.pdf]

nanomaterials

Supplementary Material

Long-Time Behavior of Surface Properties of Microstructures Fabricated by Multiphoton Lithography

Mateusz Dudziak [1,*], Ievgeniia Topolniak [1,*], Dorothee Silbernagl [1], Korinna Altmann [1] and Heinz Sturm [1,2]

[1] BAM Bundesanstalt für Materialforschung und -Prüfung, Unter den Eichen 87, 12205 Berlin, Germany; dorothee.silbernagl@bam.de (D.S.); korinna.altmann@bam.de (K.A.); Heinz.Sturm@bam.de (H.S.)
[2] TU Berlin, Institute for Machine Tools and Factory Operations (IWF), Pascalstr. 8-9, 10587 Berlin, Germany
* Correspondence: mateusz.dudziak@bam.de (M.D.); ievgeniia.topolniak@bam.de (I.T.)

AFM FDC measurement

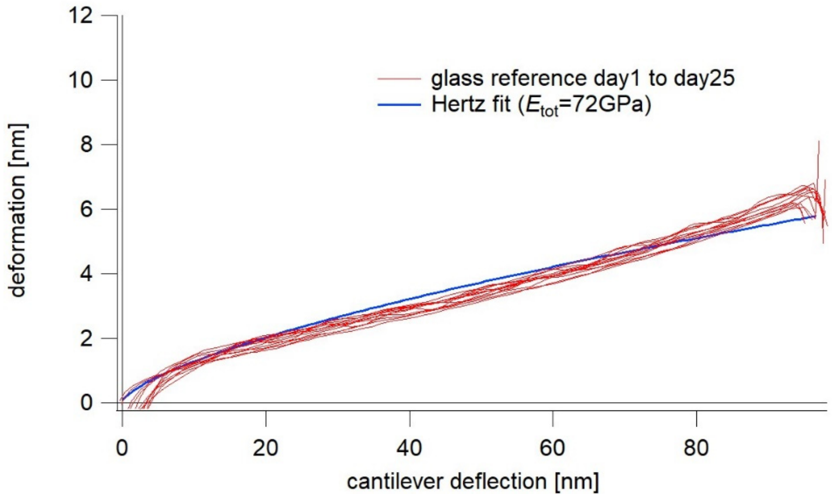

Figure S1. Averaged FDC from reference measurements on glass: curves are highly reproducible throughout the whole experiment and in very good agreement with the Hertzian fit.

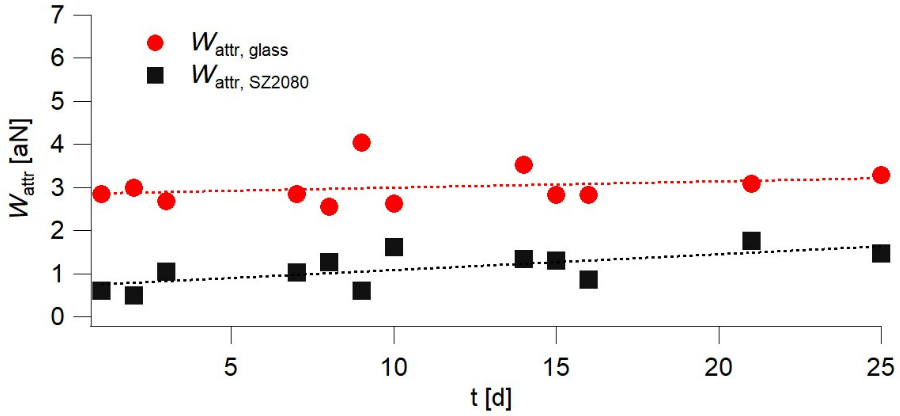

Figure S2. Comparison of work of attractive forces during approach W_{attr} of glass and SZ2080 over time. As attractive forces of glass are fairly stable, indicating a clean tip with unchanged geometry, attractive forces of SZ2080 increase, an indication of increased density of the material at the surface of MLP-fabricated structures.

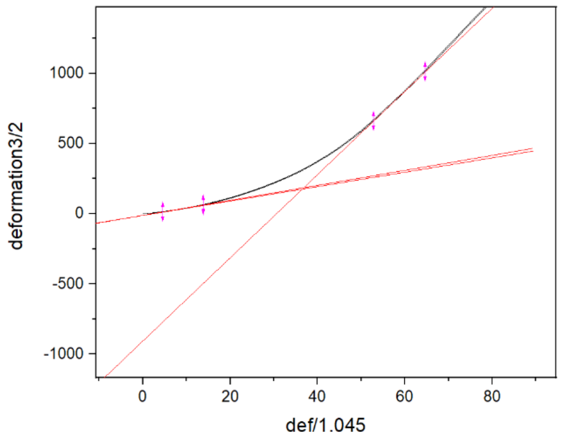

Figure S3. F_{yield} is determined by the point of intersection of linear fits of the deformation $D^{3/2}$ in the regime of elastic and plastic deformations, respectively.

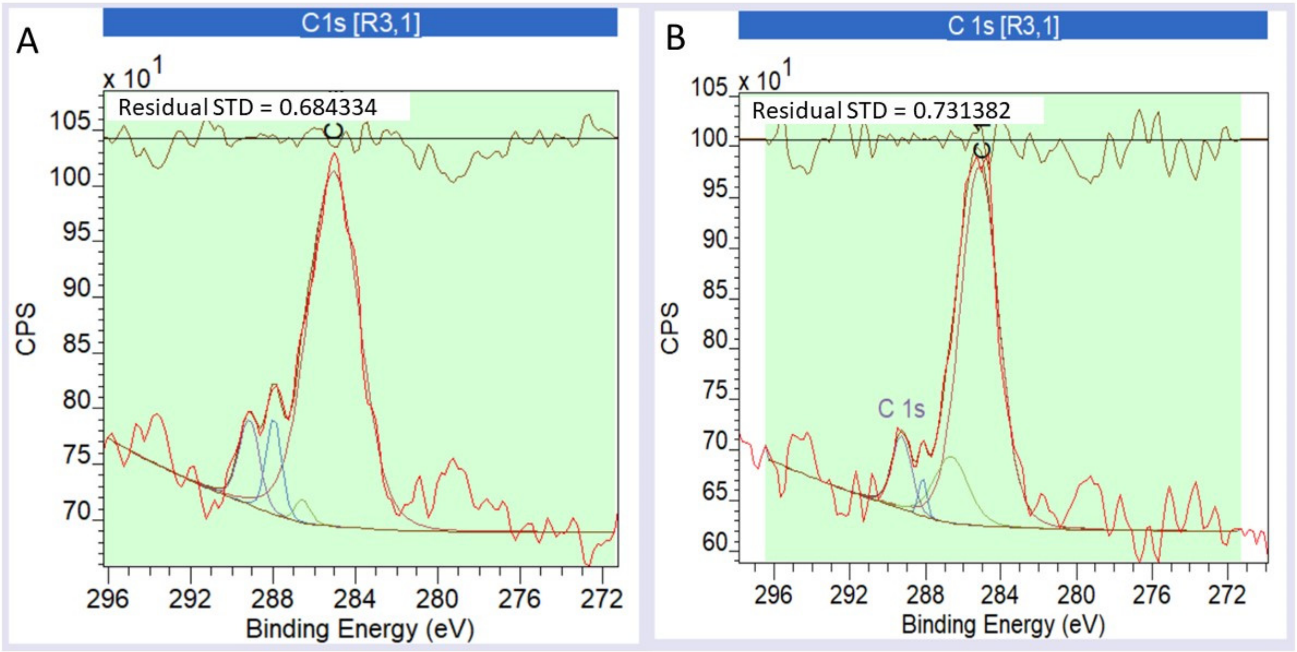

Figure S4. Deconvoluted representation of peaks C1S for freshly printed sample at day 1 (**A**) and aged sample day 25 (**B**).

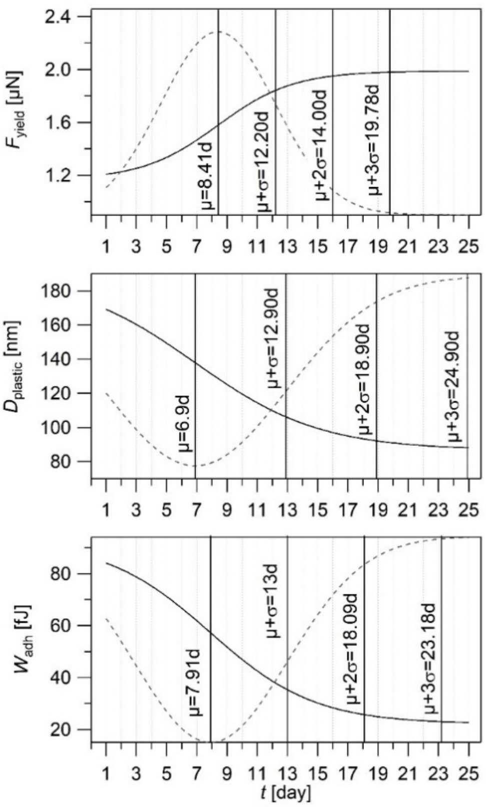

Figure S5. Statistical analysis, including the bell curve with its characteristic areas μ and μ+(n*σ).
